# Cytokine and microbiota profiles in obesity-related hypertension patients

**DOI:** 10.3389/fcimb.2023.1325261

**Published:** 2024-01-16

**Authors:** María Magdalena Aguirre-García, Amedeo Amedei, Paulina Hernández-Ruiz, Ana Pamela Gómez-García, Elena Niccolai, Aura M. Moreno-Rodríguez, Sandra Pinto-Cardoso, Adriana Alviter-Plata, Alma R. Escalona-Montaño, Erick R. Ordaz-Robles, María del C. González-Salazar, Rashidi Springall Del Villar, Enrique A. Berrios-Bárcenas, Nydia Ávila-Vanzzini

**Affiliations:** ^1^ Unidad de Investigación UNAM-INC, División de Investigación, Facultad de Medicina, Universidad Nacional Autónoma de México, Instituto Nacional de Cardiología "Ignacio Chávez, Ciudad de México, Mexico; ^2^ Department of Experimental and Clinical Medicine, University of Florence, Florence, Italy; ^3^ Interdisciplinary Internal Medicine Unit, Careggi University Hospital, Florence, Italy; ^4^ Centro de Investigación en Enfermedades Infecciosas, Instituto Nacional de Enfermedades Respiratorias "Ismael Cosío Villegas", Ciudad de México, Mexico; ^5^ Outpatient Clinic, Cardiovascular Risk Factors Clinic, Instituto Nacional de Cardiología "Ignacio Chávez", Ciudad de México, Mexico; ^6^ Department of Immunology, Instituto Nacional de Cardiología "Ignacio Chávez", Ciudad de México, Mexico

**Keywords:** human microbiome, oral microbiota, gut microbiota, obese, overweight, high blood pressure, arterial hypertension

## Abstract

**Background:**

Systemic arterial hypertension is linked to a heightened risk of cardiovascular diseases on a global scale. In Mexico, nearly half of adults in vulnerable conditions experience hypertension. Imbalance in the oral and intestinal microbiota composition has been observed in patients with hypertension, documented by a decrease of bacteria producing short-chain fatty acids, which play a critical role in blood pressure regulation.

**Aim:**

To examine the cytokines’ profile and assess the characteristics of oral and gut microbiota in obesity-related hypertension in Mexican patients.

**Methods:**

A cross-sectional, observational, and analytical study was carried out. Twenty-two patients were categorized by their body mass index (BMI) as overweight and obese, and the diagnosis of primary hypertension. DNA from supragingival dental plaque and feces samples was used to carry out 16S rRNA sequencing. Additionally, 13 cytokines were quantified.

**Results:**

In the oral microbiota, *Kluyvera* was found to be significantly enriched in obese compared to overweight patients. Instead, the gut microbiota was dominated by *Firmicutes.* However, the correlation between certain genera and proinflammatory cytokines was noted.

**Conclusion:**

This exploratory study provides insights into the complex relationship between the oral and gut microbiota and their association with systemic inflammation in obesity-related hypertension.

## 1 Introduction

Systemic arterial hypertension, a worldwide public health problem, is a pathology that can be preventable or treated to decrease premature death and disability in the population. It is associated with an increased risk of cardiovascular diseases such as coronary heart disease, stroke, chronic kidney disease, and heart failure (Unger et al., 2020). In 2015, the global prevalence was estimated at 1,130 billion people (Zhou et al., 2017), which means it affects 30 to 45% of the adult population (Chow et al., 2013), causing 10.4 million deaths per year (Mills et al., 2020). In Mexico, 49.2% of adults in vulnerable conditions suffer from hypertension (Campos-Nonato et al., 2019) and the incidence in the year 2018-2019 was 12%.

The hypertension is a multifactorial disease in which genetic and environmental factors are involved; among the causal factors are overweight and obesity, the latter increasing the incidence by 2.2 times (Campos-Nonato et al., 2018; Romero-Martínez et al., 2019). In addition, diets rich in sodium and carbohydrates contribute to the development of this disease (Jaques et al., 2021). Several authors have investigated the microbiota involvement in the development of hypertension, describing a significant decrease in the gut microbial richness and diversity compared to non-hypertensive subjects (Yang et al., 2015). Patients with high blood pressure (HBP) show a higher abundance of the genera *Klebsiella, Parabacteroides, Desulfovibrio*, and *Prevotella* in fecal samples, as well as a lower abundance of short-chain fatty acids (SCFA)-producing taxa, related to the genera *Faecalibacterium, Roseburia*, and *Bifidobacterium*, and *Ruminococcaceae* family (Verhaar et al., 2020). This holds utmost importance because SCFA play a significant role in regulating blood pressure. These metabolites are produced through the fermentation of carbohydrates that are not digested by the gut microbiota (Silva et al., 2020). Among them, acetate, propionate, and butyrate have been extensively studied. The binding of acetate and propionate to G-protein-coupled receptors, specifically GPR-43 and GPR-41, has been associated with decreased blood pressure levels in mouse models (Marques et al., 2018). Conversely, obese patients have been found to exhibit elevated levels of the *Bacteroidetes* phylum, as well as increased fecal SCFA when compared to healthy controls (Schwiertz et al., 2010; Tanaka, 2020). Studies have identified specific changes in the gut microbiota of obese patients, including an increase in the *Firmicutes* phylum, as well as the presence of genera such as *Clostridium*, and species such as *Eubacterium rectale*, *Clostridium coccoides*, *Clostridium histolyticum*, *Lactobacillus reuteri*, *Akkermansia muciniphila*, and *Staphylococcus aureus* (Gomes et al., 2018). Overall, these findings suggest that obesity-associated gut dysbiosis may contribute to increased blood pressure (Tanaka, 2020).

In addition, the dysbiosis of the oral cavity has been linked to hypertension and cardiovascular disease. Specifically, an increased abundance of bacteria such as *Aggregatibacter actinomycetemcomitans, Porphyromonas gingivalis, Tannerella forsythia, Treponema denticola*, and *Prevotella intermedia*, has been proposed to be associated with the development of systemic arterial hypertension. A study exploring the microbiota of hypertensive older women documented that *Prevotella* and *Streptococcus oralis* were present in lower abundance compared to women with normal blood pressure (Schwiertz et al., 2010; Gomes et al., 2018). Another relevant regulator of blood pressure is the nitric oxide (NO), firstly released by immune cells (Xiao and Harrison, 2020); thus, the involvement of the oral microbiota in the reduction of nitrates from the diet has been analyzed, resulting in the synthesis of nitric oxide, which is considered an important vasodilator and responsible for normal blood pressure. Among the species related to the reduction of nitrates in the oral cavity, genera such as *Veillonella, Actinomyces, Rothia*, and *Neisseria* are identified (Lamonte et al., 2022).

Likewise, some studies have identified the low-grade inflammatory state induced by obesity, finding a correlation with the BMI and pro-inflammatory cytokines such as IL-6 and TNF identified in serum of obese patients (Borges et al., 2018), which contributes as a risk factor in cardiovascular diseases (Battineni et al., 2021). The production of leptin in obese patients was identified as a pro-inflammatory factor, which promotes a TH1 response (Coppola et al., 2021); besides, the release of TNF increases leptin expression and triggers the low-grade inflammatory state (Obradovic et al., 2021). There is the knowledge that the endothelial expression VCAM-1 is inhibited by NO, and the decreases bioavailability in hypertensive patients, promote the immune cells binding to the endothelium and increases the signals of pro-inflammatory cytokines (Xiao and Harrison, 2020).

We hypothesize that dysbiosis may be related to a pro-inflammatory state influencing the high blood pressure in these patients, furthermore, the characterization of the oral (OM) and gut microbiota (GM) complex concerning obesity-related hypertension in the Mexican population, correlated with the systemic inflammatory profile, has not been described. Therefore, the aim of the present study was to examine the cytokines’ profile and assess the diversity of oral and gut microbiota in (overweight, obese I, and obese>I) patients classified, according to the blood pressure, as with systemic arterial hypertension and normal blood pressure. By knowing whether the diversity of the microbiota influence on blood pressure, weight, and inflammatory profile, we could suggest that a dietary intervention may improve their health status. After the evaluation, most of the patients were diagnosed with normal blood pressure (Non-HBP), and particularly in the overweight group, more than half of the patients were diagnosed with hypertension (HBP).

## 2 Materials and methods

### 2.1 Patients

We designed a cross-sectional, observational, and analytical study. The recruited patients underwent evaluation to assess their body mass index (BMI) and to diagnose primary systemic arterial hypertension. We excluded patients diagnosed with secondary hypertension, cancer, other chronic-degenerative diseases, or HIV/AIDS, chronic intake of alcohol or drugs, antibiotics in the 2 months prior to the study, NSAIDs, oral contraceptives, and any medication other than antihypertensive. In addition, patients with a history of gut surgery, and absorption disorders, as well as patients with serum creatinine greater than 1.3 mg/dL, pregnancy, and lactation were excluded. The present study was approved by the ethics and scientific committees of the National Institute of Cardiology (Instituto Nacional de Cardiología “Ignacio Chávez”) with No. 2018-41, and the Medicine Faculty (Facultad de Medicina, Universidad Nacional Autónoma de México) with the registration code FM/DI/030/SR/2019, according to the ethical guidelines of Helsinki and good clinical practices. After obtaining prior informed consent, patients were instructed to observe a fasting period of 12 hours. Subsequently, a comprehensive anamnesis evaluation, an oral evaluation, and an electrocardiogram were conducted for each patient.

According to the WHO parameters, patients were categorized by their BMI as overweight (25-29 kg/m^2^), obese I (30-34 kg/m^2^), and obese >I (≥ 34 kg/m^2^). Hypertension was considered according to current guidelines as high-normal: Systolic Blood Pressure (SBP)130−139 mmHg, high-normal: Diastolic Blood Pressure (DBP) 85−89 mmHg; Grade 1: SBP 140–159 mmHg, DBP 90−99mmHg; Grade 2: SBP ≥160mmHg and DBP ≥100mmHg (Unger et al., 2020). According to this guideline, the patients diagnosed with hypertension were allocated in the high blood pressure group (HBP), and those without a hypertension diagnosis in the non-high blood pressure group (Non-HBP).

### 2.2 Blood pressure measurements

The measurement was carried out in agreement with clinical practice standards using an automatic measurement system (OMRON HEALTHCARE, INC, USA; model BP710). Following 5-minute period of rest in a quiet environment with a warm temperature, the blood pressure measurement was carried out adhering to the following considerations: the patient was instructed to maintain silence, sit with an upright and supported posture, ensure both feet were correctly positioned on the floor, have an empty bladder, without having consumed caffeine or smoked in the previous 3 hours. An ideal bracelet in size was used for each patient, later the blood pressure was identified in all four limbs to rule out asymmetry; later the arm with the major blood pressure was chosen. After an additional rest period of 5 minutes, 3 measurements were made with an interval of 3 minutes between them. The final blood pressure was obtained from the average of the last two measurements.

### 2.3 Metabolic and anthropometric measurements

A blood sample of 10 ml was obtained from fasting patients to perform the measurement of serum glucose, lipid profile, complete blood biometry, blood chemistry, thyroid profile, and C-reactive protein. Samples were analyzed using standard methodologies in the Central Laboratory of the National Institute of Cardiology (Instituto Nacional de Cardiología “Ignacio Chávez”). Body conformation measurements of all patients were taken using an impedance scale (OMRON HEALTHCARE, INC, USA) which determined their weight and height. During this measurement, patients were instructed to remove their shoes and wear only a clinical gown.

### 2.4 Biological samples

a) Supragingival dental plaque: patients were advised to avoid tooth brushing, flossing, and using mouthwash for 24 hours. The plaque sample was collected by scraping the vestibular and lingual surfaces of all dental organs with a sterilized ¾ Gracey curette. The sample was transferred to a polypropylene sterile container (Eppendorf tube) with 70% ethanol and stored at -20°C until DNA extraction (Hernández-Ruiz et al., 2023).

b) Feces: a sample of approximately 5g was collected by patients in a sterile container; it was indicated to keep in refrigeration until delivery, for a maximum period of 8 hours. The samples were kept at -80°C until DNA extraction.

### 2.5 DNA extraction and 16S rRNA sequencing

The DNA extraction of supragingival plaque samples was performed using the EZ-10 Spin Column Genomic DNA Minipreps kit, Animal (Bio Basic Inc) according to the manufacturer’s instructions; otherwise, the DNA extraction of feces was performed using the QIAamp Fast DNA Stool Mini Kit (Qiagen, Hilden, Germany) following the manufacturer’s protocol. The concentration and purity were determined through the UV spectrophotometry method using the NanoDrop 2000c (ThermoFisher Scientific Waltham, MA, US). The values of A260/280 were determined for each sample considering a range between 1.8-2.0. Integrity was determined by electrophoresis in 1% agarose gel stained with ethidium bromide and visualized on a ChemiDoc MPTM UV transilluminator (Bio-Rad Hercules, CA, US). Extracted DNA samples were sent to Novogene for 16S rRNA sequencing and the libraries and sequencing were performed according to their protocols using the NovaSeq 6000 PE250 platform (Illumina). The amplified 16S rRNA region was V3-V4, and the primers and sequences used for amplification were: 341F (F: forward): 5’ CCTAYGGGRBGCASCAG-3’ and 806R (R: reverse): 5’GGACTACNNGGGTATCTAAT-3’.

### 2.6 16S rRNA data analysis

The 16S rRNA data analysis was performed using “Quantitative Insights into Microbial Ecology 2” (QIIME2) [v.2020.11]. The analysis protocol was previously described by our group (Hernández-Ruiz et al., 2023). Taxonomy of the supragingival plaque samples was assigned using the Human Oral Microbiome (eHOMD) v.15.2 databases at 99% identity pre-trained for the V3-V4 region (Escapa et al., 2018). The gut samples, as well as the complex of oral and gut niches, were analyzed using the Silva v.132 databases at 99% identity. Alpha-diversity was estimated using two metrics of Observed features and Shannon compared with the Mann-Whitney U-test; while beta-diversity was performed using the Bray-Curtis index and visualized using principal coordinate analysis. The differences between groups were assessed by Permutational Multivariate Analysis of Variance (PERMANOVA, adonis, R, 999 permutations). The differential taxonomic analysis had been performed by DESeq2 as previously described in (Curini et al., 2023).

### 2.7 Cytokines’ quantification

A panel of 13 cytokines was measured in serum samples using the LEGENDplex Human Essential Immune Response Panel kit (13plex, Biolegend). In detail, we included in our report: interleukin-4 (IL-4), IL-1β, IL-6, IL-10, tumor necrosis factor (TNF), interferon-γ (IFN-γ), and transforming growth factor-β1 (TGF-β1). The FACSAria flow cytometer from Biosciences in San Jose, CA, USA, was used for this protocol and data obtained were analyzed with the LEGENDplex Data Analysis Software Suite (https://legendplex.qognit.com).

### 2.8 Statistical analysis

Continuous variables were presented as median value and interquartile range. For the statistical analysis and graphic representation, R software [v.4.1.2] was used. Statistical analysis was performed using Student’s t-test for parametric data, as well as the Kruskal-Wallis or Mann-Whitney U-test for non-parametric values, after analyzing the distribution of data using the Shapiro-Wilk test, and Chi-square for continuous variables. A p-value ≤ 0.05 was set for significance. Correlation analysis was performed by Spearman’s rho. p-values were corrected for multiple comparisons using the Benjamini-Hochberg FDR procedure.

## 3 Results

### 3.1 Enrolled patients

We have enrolled twenty-two patients who were classified, according to their BMI, as overweight (n=10), obese I (n=5), and obese>I (n=7); according to the blood pressure, eighteen patients were diagnosed with systemic arterial hypertension at primary stage 1-2 (HBP group, 81.8%), and four had normal blood pressure (Non-HBP group, 18.2%). All the patients with normal blood pressure were overweight. Their median age was 36.3 years, with 68.2% being men and 31.8% being women. There were no significant differences observed in gender distribution between the study groups (p>0.05, Chi-square). Demographic and clinical characteristics are summarized in **Table 1**. By oral evaluation, all patients presented a DMF index (decay, missed, filled index) >6.6, considering a very high risk of caries.

**Table 1:** Clinical characteristics of patients, according to their weight status.

	Overweight n = 10	Obese In= 5	Obese>In = 7	Overalln = 22	*p-value*
**Subjects, n (%)**	10 (45.5)	5 (22.7)	7 (31.8)	22 (100.0)	
Sex					
Female	4 (18.2)	2 (9.1)	1 (4.5)	7 (31.8)	0.507 ^†^
Male	6 (27.3)	3 (13.6)	6 (27.3)	15 (68.2)	
Age					
	33.9 ± 10.2 29.5 [26.6 – 41.2]	38.0 ± 9.3 37.0 [26.5 – 49.6]	38.6 ± 8.5 42.0 [30.7 – 46.5]	36.3 ± 9.4 36.0 [27.5 – 46.3]	0.657 ^‡^
Height, mt					
	167.5 ± 7.6 169.0 [162.0 – 172.9]	164.6 ± 8.5 169.0 [154.0 – 175.2]	170.4 ± 8.9 168.0 [162.1 – 178.7]	167.8 ± 8.1 168.0 [163.0 – 171.0]	0.892 ^‡^
Weight, kg					
	78.3 ± 6.0 78.0 [74.0 – 82.6]	89.4 ± 9.0 86.0 [78.2 – 100.6]	124.7 ± 20.8 132.0 [105.4 – 144.0]	95.6 ± 24.3 85.5 [78.0 – 105.8]	> 0.0001* ^‡^
Blood Pressure					
Systolic	133.1 ± 24.1 128.5 [115.8 – 150.4]	156.4 ± 15.6 163.0 [137.0 – 175.7]	155.3 ± 15.9 152.0 [140.6 – 170.0]	145.5 ± 22.4 148.0 [129.3 - 166.5]	0.087 ^‡^
Dyastolic	94.8 ± 14.9 92.5 [84.2 – 105.5]	109.4 ± 12.5 110.0 [93.9 – 124.9]	113.1 ± 13.2 99.0 [91.0 – 115.3]	100.8 ± 14.5 99.0 [90.3 – 110.5]	
Health status					
Non-HBP	4 (18.2)			4 (18.2)	0.11^†^
HBP	6 (27.3)	5 (22.7)	7 (31.8)	18 (81.8)	
BMI					
	28.4 ± 1.6 29.2 [27.3 – 29.7]	32.6 ± 1.6 33.2 [31.6 – 33.7]	42.1 ± 6.4 39.7 [38.4 – 44.4]	33.8 ± 7.1 31.0 [29.5 – 38.0]	0.11^†^

Note.

HBP: High blood pressure; Non-HBP: Non-High blood pressure; BMI: body mass index; IQR: interquartile rank; SD: Standard deviation.

Data are indicated as number and percentage n (%), otherwise are presented as mean ± SD and median [IQR].

Non-parametric Mann-Whitney U-test was applied to compare two groups (Overweight vs Non-HBP/HBP)

The Kruskal-Wallis test was applied to compare variables with more than two groups, Overweight vs Obese I vs Obese>I.

^†^ Fisher's exact test was applied to evaluate the association between qualitative variables, and when more than 20% of cells have expected frequencies < 5; otherwise, ‡ chi-square with Yate’s correction to prevent the overestimation of statistical significance for small data when 'zero cells' are present in a 2 × 2 contingency table. The level of significance was established as *p ≤ 0.05.

### 3.2 Microbiota analysis in patients stratified according to BMI

**Figure 1:**
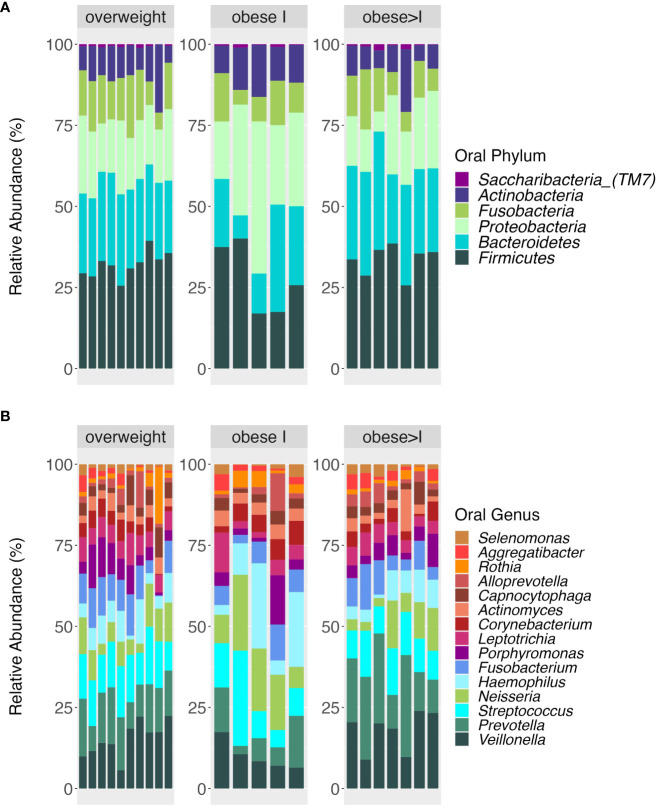
Taxonomic composition at phyla **(A)** and genera level **(B)** of oral microbiota according to the weight status (overweight, obese I, obese>I). Bar plot shows the relative abundance of bacterial taxa in each sample.

**Figure 2:**
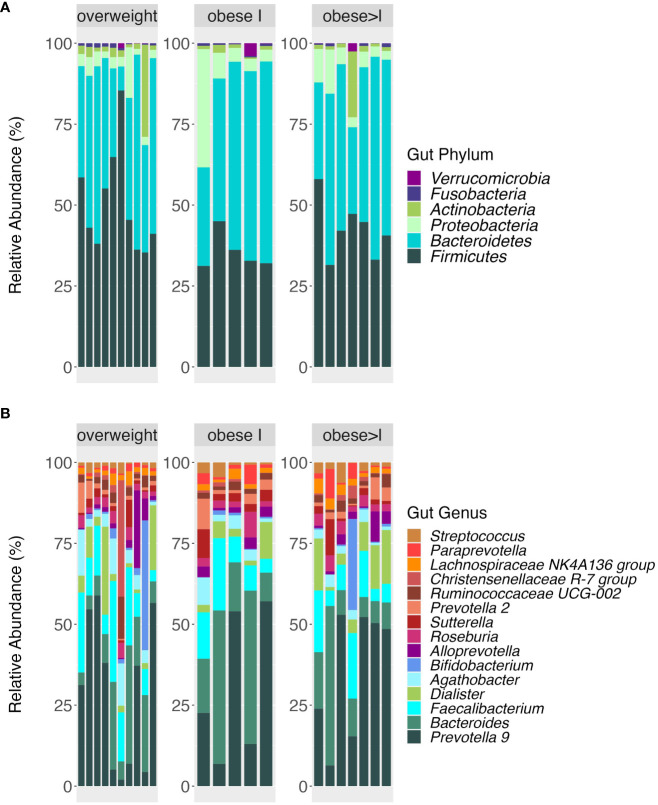
Taxonomic composition at phyla **(A)** and genera level **(B)** of gut microbiota according to the weight status (overweight, obese I, obese>I). Bar plot shows the relative abundance of bacterial taxa in each sample.

**Figure 3:**
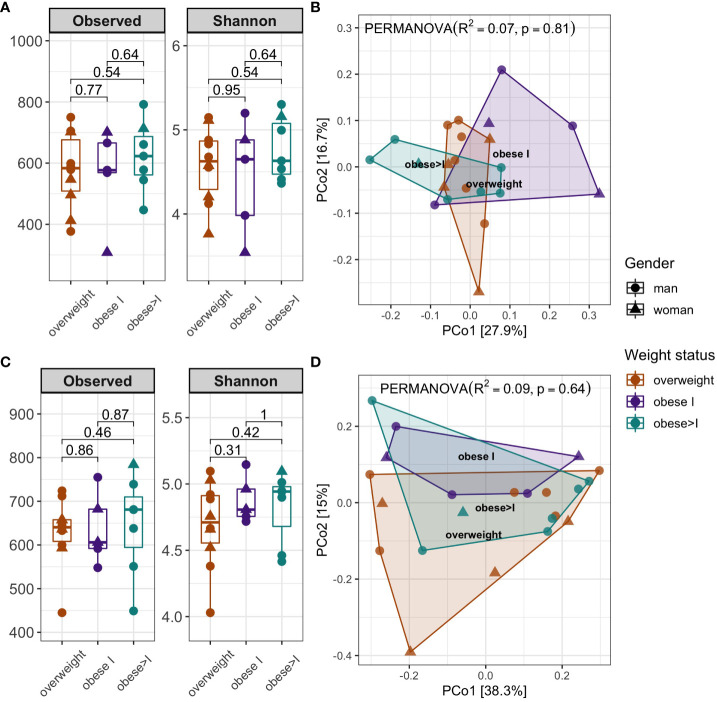
Alpha and beta diversity analysis of patients according to weight status and gender of oral **(A, B)** and gut microbiota **(C, D)**. Alpha diversity analysis was performed by Observed features and the Shannon index. Statistical differences were evaluated using Kruskal-Wallis test. P-values less than 0.05 were considered statistically significant **(A, C)**. Principal coordinate analysis of beta diversity by Bray Curtis index (PERMANOVA, R^2^, p-value) **(B, D)**.

The relative abundance at the phyla and genera level of the oral and the gut microbiota in patients divided according to their BMI are reported in **Figures 1**, **2**, respectively. First, the analysis of the alpha diversity and beta diversity of the oral (**Figures 3A, B**) and gut microbiota (**Figures 3C, D**) suggested that the overall structure of microbial communities was similar in patients with different BMI. In both sites, the five most prevalent phyla were: *Firmicutes, Bacteroidetes, Proteobacteria, Fusobacterium*, and *Actinobacteria*.

In the oral cavity, the analysis of the *Firmicutes/Bacteroidetes* ratio among the BMI categories didn´t show statistical differences, although this ratio tended to be higher in obese I patients than the other groups [1.3 (IQR= 1.05-1.7)]. In agreement, the genera level, the top three most abundant genera vary among different BMI categories. In detail, in overweight patients, the top three genera by relative abundance were as follows: *Veillonella* (13.5%), *Prevotella* (11.8%), and *Streptococcus* (11.8%). In the obese category I, they were *Neisseria* (13.2%), *Haemophilus* (11.6%), and *Streptococcus* (11.6%). Finally, for the obese>I, the most abundant genera were *Prevotella* (16.6%), *Veillonella* (15.4%), and *Streptococcus* (9.6%) (**Figure 1B**).

According to the results of the differential taxonomic analysis, in obese category >I patients, we observed significantly higher levels of two genera belonging to the *Proteobacteria* phylum when compared to overweight patients. Specifically, the genus *Kluyvera* (log2FoldChange =36.649, p.adj=1.488e-24, DESeq2), and *Yersinia* (log2FoldChange=8.390, p.adj= 4.3618e-02, DESeq2). In addition, the genus *Kluyvera* displayed significantly higher levels in obese category I when compared to overweight patients (log2FoldChange=22.432, p.adj = 6.277e-07, DESeq2).

Regarding the gut microbiota, the most abundant phylum in overweight patients was the Firmicutes (average relative abundance of 50%) whereas in obese I and obese>I patients was *Bacteroidetes* (52.9%, and 46.7% respectively) (**Figure 2A**). Analyzing the F/B ratio for the BMI categories, we reported no statistical differences, although the F/B ratio tended to be higher in overweight patients compared to the other groups [1.1 (IQR= 0.7-1.6)]. At the genus level, *Prevotella_9* was the most abundant intestinal taxa in all patients. Among overweight patients, the top three genera were *Prevotella_9* (20.6%), *Faecalibacterium* (9.4%), and *Bacteroides* (8.9%). In obese I and obese>I patients the most abundant genera were *Prevotella_9* (21.2% and 24.2%, respectively) and *Bacteroides* (18.2%, and 10.2%) (**Figure 2B**). However, no statistically significant differences were found in the GM taxonomic composition among groups.

### 3.3 Microbiota analysis in obese patients with and without hypertension

**Figure 4:**
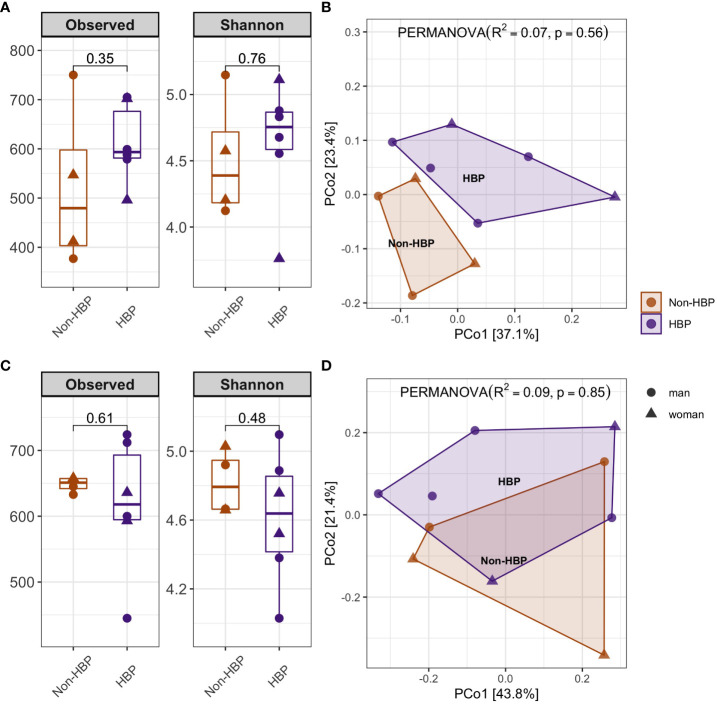
Alpha and beta diversity analysis of overweight patients according to their health status (Patients without high blood pressure “Non-HBP”, Patients with high blood pressure (“HBP”) of oral **(A, B)** and gut microbiota **(C, D)**. Alpha diversity analysis performed by Observed features and Shannon index. Statistical differences were evaluated using Mann-Whitney test. P-values less than 0.05 were considered statistically significant **(A, C)**. Principal coordinate analysis of beta diversity by Bray Curtis index (PERMANOVA, R^2^, p-value) **(B, D)**.

Next, we compared the Non-HBP (N=4) and HBP (N=6) groups within the overweight category. Regarding the alpha-diversity analysis of the oral (**Figure 4A**) and gut (**Figure 4C**) microbiota; we observed in OM a no significant higher diversity index in HBP patients in the Observed features [p=0.35] and Shannon index [p=0.76] by Mann Whitney-U test. In addition, we described in GM a no significant lower diversity in the same group in Observed features [p=0.61] and Shannon index [p=0.48] by the same test; even the beta-diversity analysis suggesting that the groups cauterize separately, but the PERMANOVA results did not show statistical differences of the oral (**Figure 4B**) and gut microbiota (**Figure 4D**).

We analyzed the F/B ratio of the oral microbiota, and the results did not show statistical differences, although there was a tendency to be higher in HBP patients than Non-HBP patients [1.3 (IQR 1.1-1.5)].

Regarding the OM composition, we found a different relative abundance of the genera *Ruminococcaceae_[G-2]* (log2FoldChange=-3.315, p.adj=0.001, DESeq2)*, Butyrivibrio* (log2FoldChange=-7.597; p.adj=0.001, DESeq2)*, Eggerthia* (log2FoldChange= -7.027, p.adj=0.004, DESeq2)*, Pedobacter* (log2FoldChange= -8.158), p.adj=0.007, DESeq2)*, Staphylococcus* (log2FoldChange= 7.955, p.adj=0.007, DESeq2) and *Fastidiosphila* (log2FoldChange= -5.887, p.adj=0.007, DESeq2) in HBP compared to Non-HBP patients. Instead for the GM composition and the F/B ratio (data not shown), we didn’t document any statistical difference between Non-HBP and HBP patients.

**Figure 5:**
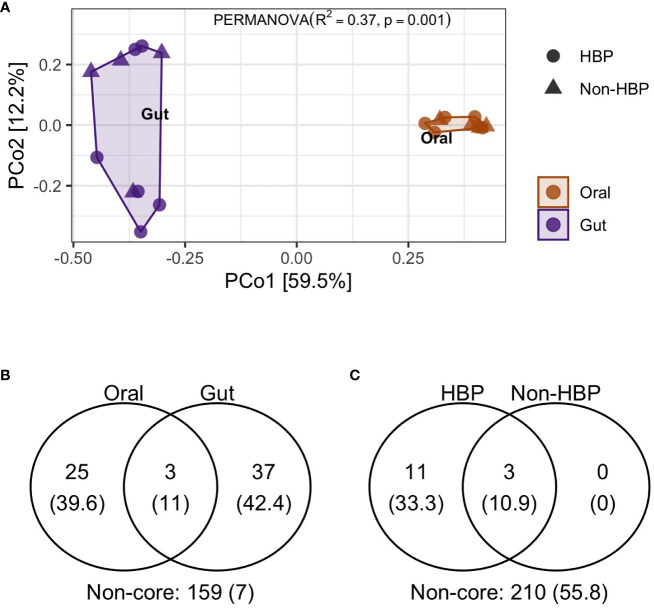
Oral and gut microbiota diversity of overweight patients according to their health status (Patients without high blood pressure “Non-HBP”, Patients with high blood pressure (“HBP”). **(A)** Principal coordinate analysis of beta diversity byBray Curtis index (PERMANOVA, R^2^, p value) identified in the oral and gut microbiota in Non-HBP and HBP patients. **(B)** Venn diagram of oral and gut taxa of overweight patients [number of OTU (%)]. **(C)** Venn diagram of overweight samples in Non-HBP and HBP patients [number of OTU (%)].

Lastly, in the same patients we compared the oral and gut microbiota composition and as expected, the PCoA analysis displayed distinct clustering (**Figure 5**), suggesting that the variance among both niches was determined to be 37% (R^2 =^ 0.37, p=0.001) (**Figure 5A**). Interestingly, the Venn diagrams (**Figure 5B**) revealed the presence of three genera shared in both niches, namely *Prevotella_2, Alloprevotella, Streptococcus*. Conversely, we identified 11 genera specific for HBP patients (*Dialister, Veillonella, Corynebacterium, Actinomyces, Leptotrichia, Capnocythophaga, Bacteroides, Prevotella, Prevotella_7, Porphyromonas*, and *Fusobacterium*) (**Figure 5C**).

### 3.4 Serum cytokines profiling

**Figure 6:**
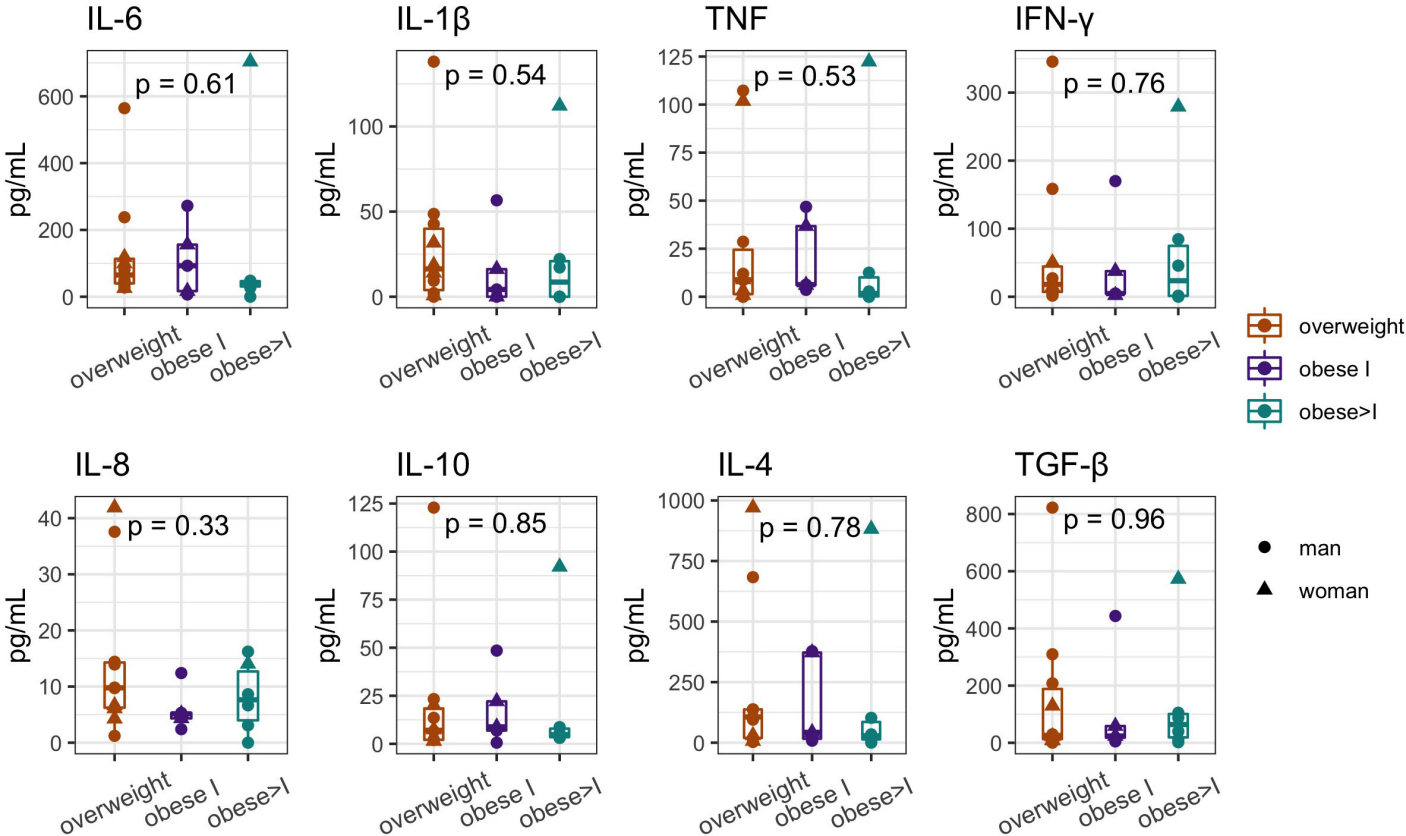
Cytokines’ serum concentration (median and interquartile range; pg/ml) in overweight, obese I, obese>I patients; showing the Statistical differences were evaluated using the Kruskal Wallis test. P-values less than 0.05 were considered statistically significant.

Compared to other groups, the overweight patients seems to have higher concentration (pg/ml) of different evaluated cytokines, in detail IL-1β [16.3 (2.10- 42.68)], IL-8 [9.8 (6.09-14.41)], IL-4 [106.0 (15.96- 137.52)] and TGB-β [25.9 (10.40-207.90)]; whereas the obese >I patients, showed a trend towards higher concentration of IL-6 [42.2 (27.05-47.95)], TNF [1.72 (0-12.52)]; IFN-γ [23.53 (1.18-84.49)] and IL-10 [4.75 (3.31-8.69)]. However, we did not document any significant differences among the studied groups (**Figure 6**). Additionally, no difference was observed among Non-HBP and HBP patients (**Supplementary Table 1**).

### 3.5 Correlation of cytokines and microbiome

**Figure 7:**
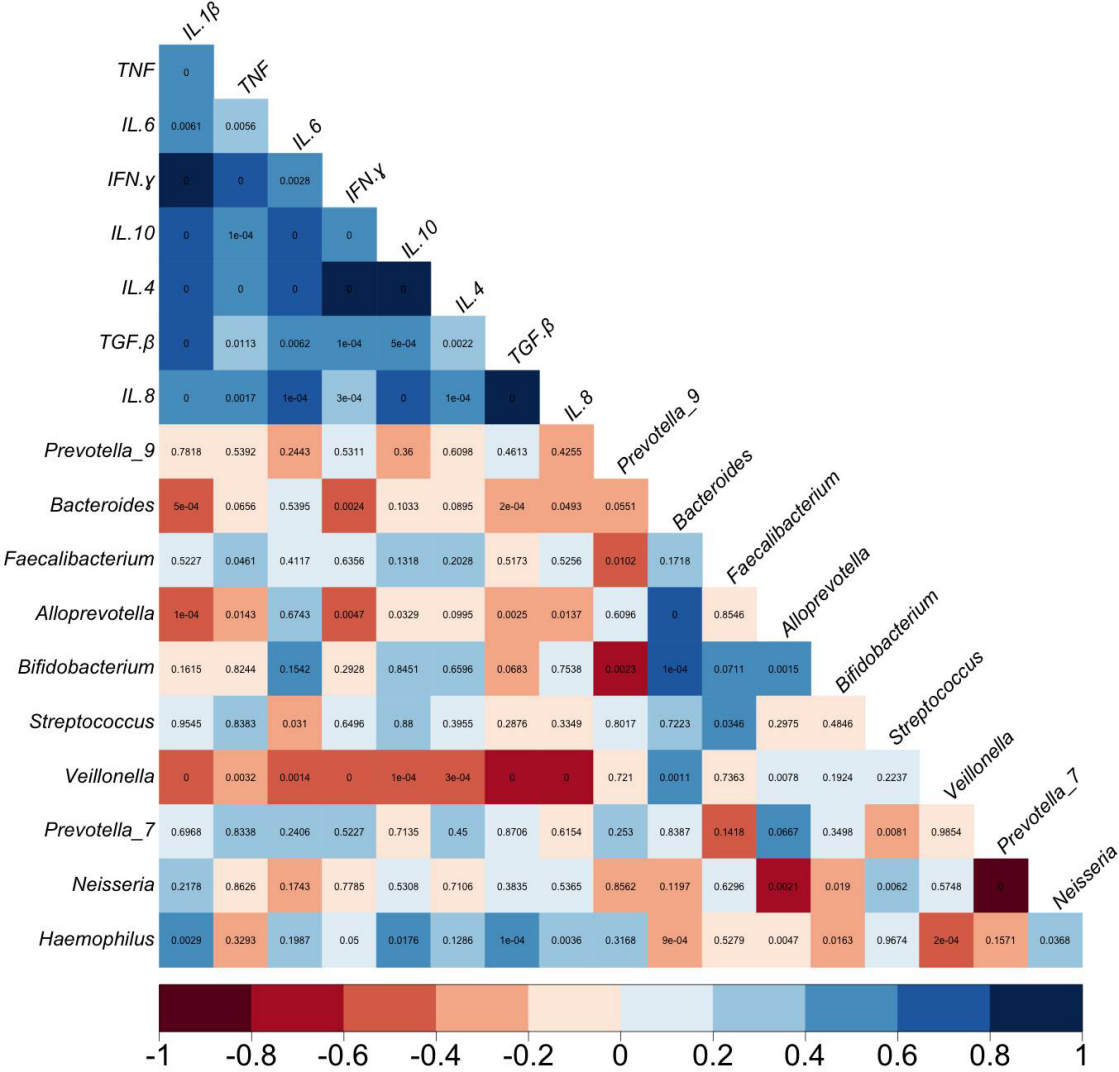
Heatmap of correlation between cytokines levels and the five most abundant taxa resulted in oral (*Streptococcus, Veillonella, Prevotella_7, Neisseria, Haemophilus*) and gut (*Prevotella_9, Bacteroides, Faecalibacterium, Alloprevotella, Bifidobacterium*) microbiota in overweight patients. Rho Spearman, p.adjust value, Benjamini Hochberg.

In the correlation analysis with the Spearman’s rank test adjusted for multiple comparison by Benjamini Hochberg, we examined the relationship between the five most abundant genera found in the oral cavity (*Streptococcus*, *Veillonella*, *Prevotella*_7, *Neisseria*, and *Haemophilus*) and in the gut (*Prevotella*_9, *Bacteroides*, *Faecalibacterium*, *Alloprevotella*, and *Bifidobacterium*) and the tested cytokines’ levels (**Figure 7**). Regarding the GM, the analysis revealed that *Bacteroides* correlated negatively with IL-1β (r [-.73], p.adj = 0.0005) and IFN-γ (r [-.66], p.adj = 0.002), while *Alloprevotella* correlated negatively with IL-1β (r[-.78], p.adj= 0.0001), and IFN-γ (r[-.65], p.adj = 0.004).

In addition, the oral genus *Streptococcus* correlated negatively with IL-6 (r [-.50], p.adj = 0.03), while *Veillonella* correlated negatively with IL-6 (r[-.69], p.adj = 0.001), TNF (r[-.65], p.adj = 0.003), also with IL-10 (r [-.80], p.adj = 0.00006), and IL-4 (r[-.74], p.adj = 0.0003). Finally, we found a positive correlation of *Haemophilus* with IL-1β (r[.69], p.adj = 0.002), with TGF-β (r[.80], p.adj = 0.00005) and IL-8 (r[-.64], p.adj = 0.003) (**Figure 7**).

## 4 Discussion

In this study, we investigated the oral and gut microbiota composition as well as the serum cytokines’ profile in Mexican patients with and without hypertension, stratified according to their BMI category. Understanding the interplay between the microbiota and cytokines’ signatures in the context of hypertension and obesity may offer valuable insights into the pathophysiology of these conditions.

In agreement with previous studies (Dewhirst et al., 2010) in the OM composition, the most abundant phyla were *Firmicutes, Bacteroidetes, Proteobacteria, Fusobacteria*, and *Actinobacteria.* OM dysbiosis has been associated with various oral (such as gingivitis, and periodontitis) and systemic diseases, including cardiovascular disease (Sohail et al., 2021). Furthermore, the presence of *Firmicutes*, and especially the genera *Granullicatella* and *Streptococcus*, in the oral cavity has been positively correlated with BMI, poor oral hygiene, and obesity. Interestingly, both genera were identified in our study cohorts, being among the 25 most prevalent types of bacteria in the oral cavity (Raju et al., 2019). In addition, the enrolled patients displayed gingivitis and high risk of caries, in that case, we suggest that the poor oral hygiene are related to the microbiota diversity. The most frequent genera identified in oral microbiota were *Veillonella, Prevotella, Streptococcus, Neisseria, Haemophilus*, and *Fusobacterium* which have been previously classified as part of the “core saliva microbiome” (Goh et al., 2019; Yang et al., 2019; Yang et al., 2019; Kasai et al., 2015).

According to studies, obesity or a predisposition to weight gain is connected to an increase in *Actinomyces*, *Aggregatibacter*, and *Firmicutes* and a decrease in *Bifidobacterium* and *Lactobacillus* in the oral microbiota (Rosing et al., 2017; Raju et al., 2019; Sohail et al., 2021); indeed, in the present cohort, the PERMANOVA analysis of beta diversity showed that the degree of obesity accounted for 7% of the variance in the oral samples. Morover, we identified *Prevotella* as the most abundant genus in obese>I patients, as shown in other analysis in obese patients that reported a high abundance of periodontal pathogens (Tribble et al., 2019; Maciel et al., 2016). We observed that the genus *Kluyvera* was enriched in obese>I compared to overweight patients. There have been reports about the pathogenic activity and antibiotic resistance of the genus *Kluyvera*, although it is generally considered as commensal bacteria in respiratory, urinary, and intestinal tract (Lee et al, 2019). Interestingly, in gut samples, *Kluyvera* was found to correlate negatively with 25-hydroxy vitamin D in obese women with polycystic ovary syndrome (Bai et al., 2023), highlightening the potential significance of *Kluyvera* in different disease contexts and warranting further investigation into its role in obesity-related conditions. Finding genus *Kluyvera* enriched in the group of Mexican patients with in obese>I, it is interesting to expand its study.

In agreement with previous studies, we reported no significant difference in comparing alpha and beta diversity between hypertensive patients and controls (Goh et al., 2019; Morou-Bermúdez et al., 2022). However, we observed a trend towards greater abundance and richness in overweight HBP patients.

Furthermore, in HBP patients, we found an increase in the genera *Ruminococcaceae_G_2, Eggerthia*, and *Staphylococcus*, all belonging to the *Firmicutes* phylum. This finding is consistent with the cohort analyzed by Sohail et al., where the phylum *Firmicutes* was also identified with a higher abundance in patients with arterial hypertension (Sohail et al., 2021). These similarities in the microbial profiles between our study and previous data highlight the potential role of the *Firmicutes* phylum in hypertension, suggest its relevance in understanding the relationship between the gut microbiota and cardiovascular health.

Of note, *Prevotella_9* was the most abundant genera found in the oral microbiota of enrolled patients. *Prevotella* genus is considered an opportunistic pathogen in the development of periodontal diseases, and many studies found a positive correlation between *Prevotella*, blood pressure, and glucose levels in adults, as well as an association with adolescent overweight s (Yang et al., 2019; Kasai et al, 2015). Additionally, *Prevotella* and *Streptococcus* have been linked to the increase in diastolic blood pressure in post-menopausal stage women (Goh et al., 2019; Morou-Bermúdez et al., 2022).

The significance of the relationship between oral dysbiosis and hypertension risk was documented in subjects who were prescribed oral antiseptics containing chlorhexidine. The use of these antiseptics led to a greater abundance of *Prevotella* and *Leptotrichia* in the oral microbiota, which was associated with a decreased reduction in nitrates, which could potentially lead to an increase in blood pressure (Tribble et al., 2019).

Regarding the gut microbiota, several studies have related the intestinal *Firmicutes/Bacteroidetes* ratio alteration with the development of some systemic diseases, including obesity (Goh et al., 2019; Raju et al., 2019). In our study, the analysis of the F/B ratio didn´t show any statistical differences, although it tended to be higher in overweight patients than the other BMI groups. The gut is a niche with extensively characterized microbiota, where the four most abundant *phyla* are *Firmicutes, Bacteroidetes, Actinobacteria*, and *Proteobacteria* (Maciel, et al., 2016; Cresci and Izzo, 2019; Chen et al., 2023). In the present study, the phylum *Firmicutes* was the most abundant phyla in overweight patients, with a relative frequency greater than % in the remaining patients. Besides, the enrichments of some genera belonging to *Firmicutes*, such as *Lactobacillus* and *Streptococcus* have been positively correlated with BMI in other studies, we identified the *Streptococcus* among the 25 most prevalent genera with similar frequency values in the different patients’ subgroups. A probable explanation could be that both groups consisted of overweight patients.

Based on the alpha and beta diversity analysis results, the GM composition did not show significant differences among patients sub-grouped by both BMI and blood pressure.

Previous studies on the gut microbiota of patients with hypertension have reported a lower alpha diversity, suggesting gut dysbiosis. This trend was observed also in our study, where, even if not significantly, HBP patients showed a lower richness and abundance compared to non-HBP patients. A similar finding was reported in a previous Finland study (Mushtaq et al., 2018), suggesting that in the presence of two or more multifactorial diseases, the level of diversity may not necessarily be lower when compared to groups that only have one pathology. In the case of the beta diversity analysis, we did not document significant results regarding the different patients’ groups.

The composition of OM and GM in overweight patients has an approximate similarity of 37%, as reported in previous findings (Kaplan et al., 2020; Li et al., 2021). However, they differ in the abundance of some bacterial species between the two niches. Notably, some oral species have been detected in stool samples of humans, with *Streptococcus* and *Prevotella* being associated with the development of colorectal cancer (CRC). Along with these genera, *Peptostreptococcus*, *Parvimonas*, and *Fusobacterium* were identified in healthy subjects. (Flemer et al., 2018; Li et al, 2021). Likewise, in CRC patients, it was documented that identical strains of *Fusobacterium nucleatum* were identified in both niches, suggesting that oral species can survive the acidic environment of the stomach and reach the gut, which may play a role in CRC development (Flemer et al., 2018; Komiya et al., 2019). Also, in a cohort of healthy adults (average age of 35 years), have been revelated 14 shared taxa between the samples of subgingival plaque and feces, and some of them could not be identified in the database of the human oral microbiome (Iwauchi et al., 2019; Komiya et al., 2019). In our current analysis, we found three genera that are shared between the oral and gut microbiota of overweight patients. This finding aligns with previous studies that also identified similar genera, such as *Streptococcus* and *Prevotella*. Additionally, we observed an increased abundance of species from the *Bacteroidetes* phylum in the overweight patients’ microbiota. These results suggest potential interactions and similarities between the oral and gut microbiota in individuals with higher BMI.

Inflammatory cytokines play a crucial role in obesity and arterial hypertension, as well as in other diseases. Chronic intestinal diseases such as Crohn’s disease and ulcerative colitis are partially attributed to an increase in TNF and IL-1β (Lacruz-Guzmán et al., 2013). In overweight Non-HBP patients, we documented a slight increase in pro-inflammatory (such as TNF, IL-6, and IL-1β) and regulatory (including IL-10, IL-4, and TGF-β) cytokines compared to HBP patients. Indeed, the increase in proinflammatory cytokines has been well-documented in overweight and obese individuals. Adipose tissue, especially in the context of excess fat accumulation, is known to release a variety of proinflammatory cytokines into the systemic circulation (Wang and He, 2018).

Indeed, the negative correlation observed between proinflammatory cytokines (IL-6, IL-1β, TNF, and IFN-γ) and certain genera of gut bacteria (*Bacteroides*, *Alloprevotella*, and *Veillonella*) in overweight individuals suggests a potential link between gut dysbiosis and inflammation in the context of weight status.

In addition, it is well known that gut microbiota impacts maintaining immune homeostasis, influencing the host’s inflammatory response. A healthy GM is associated with the production of anti-inflammatory cytokines and a balanced immune system. While a taxonomic or functional dysbiosis has been associated with increased inflammation and immune dysfunction (Niccolai et al., 2020).

In overweight individuals, dysbiosis may lead to an altered microbial profile, characterized by changes in the abundance of specific genera such as *Bacteroides*, *Alloprevotella*, and *Veillonella*. This dysbiosis may influence the production of proinflammatory cytokines, including IL-6, IL-1β, TNF, and IFN-γ. On the other hand, the positive correlation observed between *Haemophilus* and proinflammatory cytokines (IL-1β, TGF-β, and IL-18) suggests that this genus might be associated with an inflammatory response in overweight individuals.

Overall, these findings highlight the intricate relationship between the gut microbiota and the immune system in the context of weight status. Dysbiosis may lead to an inflammatory state, playing a role in the development or progression of metabolic disorders, including obesity-related hypertension. Further research is needed to fully understand the mechanisms underlying this association and to explore potential therapeutic interventions targeting the gut microbiota to mitigate inflammation and its detrimental effects in overweight individuals.

Surely, the present study shows some limitations that need to be acknowledged. Firstly, the number of enrolled patients is relatively small, which might limit the statistical power to detect significant differences, especially in subgroup analyses. A larger sample size would have provided more robust and reliable results, allowing for a more comprehensive assessment of the relationship between the oral and gut microbiota and hypertension in different BMI categories. Secondly, the study focused on Mexican patients, and the findings may not be readily applicable to different ethnic or geographical populations. The impact of ethnic and environmental factors on GM composition is well-documented, and further studies in diverse populations are necessary to better understand the global relevance of the findings. Furthermore, the cross-sectional design of the study limits our ability to establish causal relationships. It is hard to define if the observed differences in the microbiota are a cause or a consequence of hypertension and obesity. Longitudinal studies would be more suitable for assessing the temporal relationship between the microbiota and health outcomes. Lastly, although the study considered relevant confounding factors such as age and gender, there may still be other unmeasured confounders that could influence the results. Diet, physical activity, and medication use, among other factors, can influence the gut microbiota and should be considered in future investigations.

## 5 Conclusion

This is the first study where two microbial niches are simultaneously analyzed, and weight status is compared in hypertensive and non-hypertensive Mexican patients. It is important to remark that all patients presented some degree of overweight or obesity, considered as a chronic inflammatory state and allowed us to elucidate that the variability in the diversity of the microbiota among these patients is primarily influenced by body weight. Additionally, through a comparison of the composition of both environments, we were able to detect the migration of genera from the oral cavity to the intestine. This finding underscores the significance of the oral-gut axis and how it is impacted by the dysbiosis observed in these patients.

## Data availability statement

The datasets presented in this study can be found in online repositories. The names of the repository/repositories and accession number(s) can be found in the article/**Supplementary Material**.

## Ethics statement

The studies involving humans were approved by the ethics and scientific committees of the National Institute of Cardiology (Instituto Nacional de Cardiología “Ignacio Chávez”) with No. 2018-41, and the Medicine Faculty (Facultad de Medicina, Universidad Nacional Autónoma de México) with the registration code FM/DI/030/SR/2019. The studies were conducted in accordance with the local legislation and institutional requirements. The participants provided their written informed consent to participate in this study. Ethical approval was not required for the study involving animals in accordance with the local legislation and institutional requirements because the present study involves research on humans.

## Author contributions

MMA-G: Conceptualization, Funding acquisition, Project administration, Writing – original draft. AA: Formal Analysis, Supervision, Writing – review & editing. PH-R: Data curation, Formal Analysis, Investigation, Methodology, Visualization, Writing – original draft. AG-G: Investigation, Writing – original draft. EN: Formal Analysis, Supervision, Writing – review & editing. AM-R: Investigation, Writing – original draft. SP-C: Data curation, Formal Analysis, Methodology, Writing – review & editing. AA-P: Data curation, Formal Analysis, Investigation, Methodology, Visualization, Writing – original draft. AE-M: Formal Analysis, Investigation, Methodology, Supervision, Writing – original draft. EO-R: Investigation, Writing – original draft. MG-S: Methodology, Writing – review & editing. RS: Methodology, Writing – review & editing. EB-B: Methodology, Writing – review & editing. NÁ-V: Conceptualization, Project administration, Writing – original draft.
