# Supplementary material for: Cytokine and microbiota profiles in obesity-related hypertension patients
Source: Front Cell Infect Microbiol. 2024 Jan 16;13:1325261. doi: 10.3389/fcimb.2023.1325261 (PMC10824975; doi:10.3389/fcimb.2023.1325261)
Supplement: Supplementary file 1 [file Table_1.docx]

|  | **Total**  **Overweight (n=10)**  **pg/mL** | **Overweight**  **Non-HBP**  **(n=4)**  **pg/mL** | **Overweight**  **HBP**  **(n=6)**  **pg/mL** | **p value** |
| --- | --- | --- | --- | --- |
| **IL 6** | 49.7(27.5-118.1) | 74.5(43.2-108.7) | 65.0 (1.5-237.9) | 0.698 |
| **IL 1β** | 14.0(0-31.6) | 25.1(9.2-37.1) | 11.8 (2.0-48.6) | 0.574 |
| **TNF** | 6.2(0.8-28.6) | 20.2(10.4-65.2) | 1.9(0-8.6) | 0.120 |
| **IFN γ** | 14.4(2.4-50.1) | 35.9(11.6-104.3) | 13.8(5.0-27.1) | 0.635 |
| **IL 8** | 6.6(4.3-13.9) | 11.8(8.1-27.9) | 7.9(4.2-14.4) | 0.965 |
| **IL 10** | 6.9(3.0-20.0) | 7.0(4.3-13.7) | 7.8(1.4-23.2) | 0.172 |
| **IL 4** | 42.5(15.9-137.5) | 127.0(59.9-553.8) | 62.7(15.9-137.5) | 0.517 |
| **TGF β** | 29.6(11.8-129.3) | 74.7(14.0-219.4) | 25.9(10.4-207.9) | 0.897 |

**Supplementary table 1**. Cytokines’ levels (pg/ml) of overweight patients according to their health status (Patients without high blood pressure “Non-HBP”, Patients with high blood pressure “HBP”); showing the median and interquartile range calculated by Tukey range test. p value, Mann-Whitney test.
